# Complete Genome Sequence of *Sphingopyxis macrogoltabida* Strain 203N (NBRC 111659), a Polyethylene Glycol Degrader

**DOI:** 10.1128/genomeA.00529-16

**Published:** 2016-06-09

**Authors:** Yoshiyuki Ohtsubo, Shouta Nonoyama, Yuji Nagata, Mitsuru Numata, Keiko Tsuchikane, Akira Hosoyama, Atsushi Yamazoe, Masataka Tsuda, Nobuyuki Fujita, Fusako Kawai

**Affiliations:** a Department of Environmental Life Sciences, Graduate School of Life Sciences, Tohoku University, Sendai, Japan; b Biological Resource Center, National Institute of Technology and Evaluation, Tokyo, Japan; c Center for Fiber and Textile Science, Kyoto Institute of Technology, Kyoto, Japan

## Abstract

We determined the complete genome sequence of *Sphingopyxis macrogoltabida* strain 203N, a polyethylene glycol degrader. Because the PacBio assembly (285× coverage) seemed to be full of nucleotide-level mismatches, the Newbler assembly of MiSeq mate-pair and paired-end data was used for finishing and the PacBio assembly was used as a reference. The PacBio assembly carried 414 nucleotide mismatches over 5,953,153 bases of the 203N genome.

## GENOME ANNOUNCEMENT

*Sphingopyxis macrogoltabida* strain 203 was isolated from soil as the polyethylene glycol (PEG)-utilizing *Flavobacterium* sp. strain 203 (1). Later, the strain was designated the type strain of *Sphingomonas macrogoltabidus* (2) and reidentified as *Sphingopyxis macrogoltabida* (3), based on the taxonomical standards proposed by Yabuuchi et al. (4). The strain was deposited to the National Institute of Technology and Evaluation (Tokyo, Japan) and stocked under the number NBRC 15033. The complete genome of NBRC 15033 was determined, but the genes for PEG utilization were missing, and repeated cultivation was assumed to be the reason for the loss (5). From a laboratory stock, we recovered a strain, designated 203N, harboring the *pegA* gene (6, 7) and capable of growing on PEG.

Here, we report the complete genome sequence of *S. macrogoltabida* 203N. To determine the complete sequence, we obtained PacBio data from Macrogen Japan. The total number of reads obtained was 237,846 with an *N*_50_ length of 9,733 bp and a total length of 1.7 Gb. The reads were assembled by HGAP3, and three circular contigs corresponding to the main chromosome and two plasmids were obtained. However, we found that the sequences differ considerably to those of NBRC 15033 (5). Besides one genomic rearrangement, which was predicted to cause the loss, and 10 differences related to insertion sequences, a huge number (approximately 400) of nucleotide-level mismatches were counted. We also obtained Illumina MiSeq reads from the very DNA solution used for PacBio sequencing, and the assembled contig sequences suggested that PacBio assembly was erroneous. Replacing each part of the PacBio assembly by a corresponding MiSeq contig seemed inappropriate for correcting the errors, because the nucleotide-level mismatches were located throughout the genome, and contigs deriving from repeats in the genome might carry variation bases. Therefore, we decided to start the finishing from the Newbler assembly of the MiSeq reads obtained from mate-pair and PCR-free paired-end libraries. The finishing was facilitated by using ShortReadManager, GenoFinisher, and AceFileViewer (AFV) (8), which have been used to determine complete genome sequences, often enabling the complete *in silico* finishing, especially when the PCR-free kit of the Illumina sequence library preparation was used. In the finishing, the PacBio assembly was used as a reference to search the correct paths of contigs that fill each gap in scaffolds. The correct sequences of the paths were determined by AFV. The finished sequence was confirmed by FinishChecker, wherein genomic *k*-mers not found in the MiSeq reads were searched and corrected as necessary. Thus, the complete genome sequence of 203N was determined. We found 414 nucleotide-level mismatches to the PacBio assembly, most of which were found at homopolymeric stretches, which is not a characteristic error pattern of Illumina sequencing reads but may be one for PacBio.

The sequences were annotated by the NCBI Prokaryotic Genome Annotation Pipeline (PGAP) and curated using GenomeMatcher (9). While referring to the annotation data obtained from the Microbial Genome Annotation Pipeline (http://www.migap.org) (10), we corrected start codon positions and added genes that were missing in the PGAP annotation.

### Nucleotide sequence accession numbers.

The genome sequence of *Sphingopyxis macrogoltabida* strain 203N has been deposited in NCBI/GenBank under the accession numbers CP013344 to CP013346. *Sphingopyxis macrogoltabida* strain 203N is available from the Biological Resource Center, National Institute of Technology and Evaluation (Tokyo, Japan). Its deposit number is NBRC 111659.
